# Revolutionizing the understanding of acupoint biology and acupuncture mechanisms through spatial omics: current perspectives and future directions

**DOI:** 10.3389/fmed.2026.1863599

**Published:** 2026-07-21

**Authors:** Xinzhi Guo, Jing Cao, Taohong Su, Xinxin Ding, Aolin Chen, Benson Kiprono Kosgei, Delin Zhang, Fang Wang, Ping Luo

**Affiliations:** 1 The Jiangxi Province Key Laboratory for Diagnosis, Treatment, and Rehabilitation of Cancer in Chinese Medicine, Mass Spectrometry Research Center for Diagnosis, Treatment, and Chronic Disease Rehabilitation, Jiangxi University of Chinese Medicine, Nanchang, Jiangxi, China; 2 College of Traditional Chinese Medicine, Jiangxi University of Chinese Medicine, Nanchang, Jiangxi, China; 3 Institute of Traditional Chinese Medicine and Health Development, Jiangxi University of Chinese Medicine, Nanchang, Jiangxi, China

**Keywords:** acupoint biology, acupuncture, molecular medicine, spatial molecular architecture, spatial omics, systemic regulatory networks

## Abstract

Characterizing how localized physical stimulation at acupoints elicits systemic therapeutic effects is essential to understanding acupuncture biology. Spatial omics technologies offer a transformative approach to this challenge by enabling high-resolution, multiplexed analysis of biomolecules directly within their native tissue contexts, thereby decoding the spatial molecular architecture of acupoints and mapping the systemic regulatory networks underlying acupuncture effects. This review synthesizes the spatial and anatomical properties of acupoints and the established molecular mechanisms of acupuncture, focusing on key receptors and signaling pathways. We further provide a comprehensive overview of spatial omics technologies and their groundbreaking applications in both Western and Chinese medicine. Critically, we explore the potential of spatial omics to revolutionize acupuncture research by delineating the spatial biological architecture of acupoints, visualizing the local tissue response to stimulation, and ultimately deciphering the spatiotemporal dynamics of the remote network responses that underpin acupuncture’s systemic effects. Overcoming current technical and analytical challenges will be pivotal in harnessing spatial omics to investigate the proposed spatial principles of acupuncture, thereby bridging a critical gap between its empirical practice and mechanistic understanding.

## 1 Introduction

Acupuncture, a core external treatment modality in Traditional Chinese Medicine (TCM), has been practiced for over two millennia. This ancient therapy demonstrates unique advantages in being green and safe, offering holistic regulation, and enabling individualized treatment. Consequently, the World Health Organization recommends acupuncture for treating 107 conditions across seven major categories, including pain, neurological disorders, and gastrointestinal dysfunction (1). The fundamental principle of acupuncture lies in the biological effects generated by mechanical stimulation signals when needles activate specific points on the human body (2). However, how this micrometer-scale needle stimulation triggers cross-scale, cross-organ therapeutic cascades remains an enigma (3).

Living organisms are highly organized spatial systems whose functionality depends on the precise coordination of multiscale three-dimensional structures, progressing from molecules (nanoscale) to cells (micrometer scale), then to tissues and organs (millimeter-centimeter scale), and finally to the entire organism (meter scale) (4). In this context, acupuncture is considered a therapeutic intervention that employs holistic regulation, making it a clear manifestation of spatial-scale modulation. However, conventional acupuncture mechanism studies, predominantly relying on non-spatial omics approaches, can only analyze homogenized tissue samples, making it difficult to uncover molecular spatial characteristics at the microscopic level of acupoints.

**FIGURE 1:**
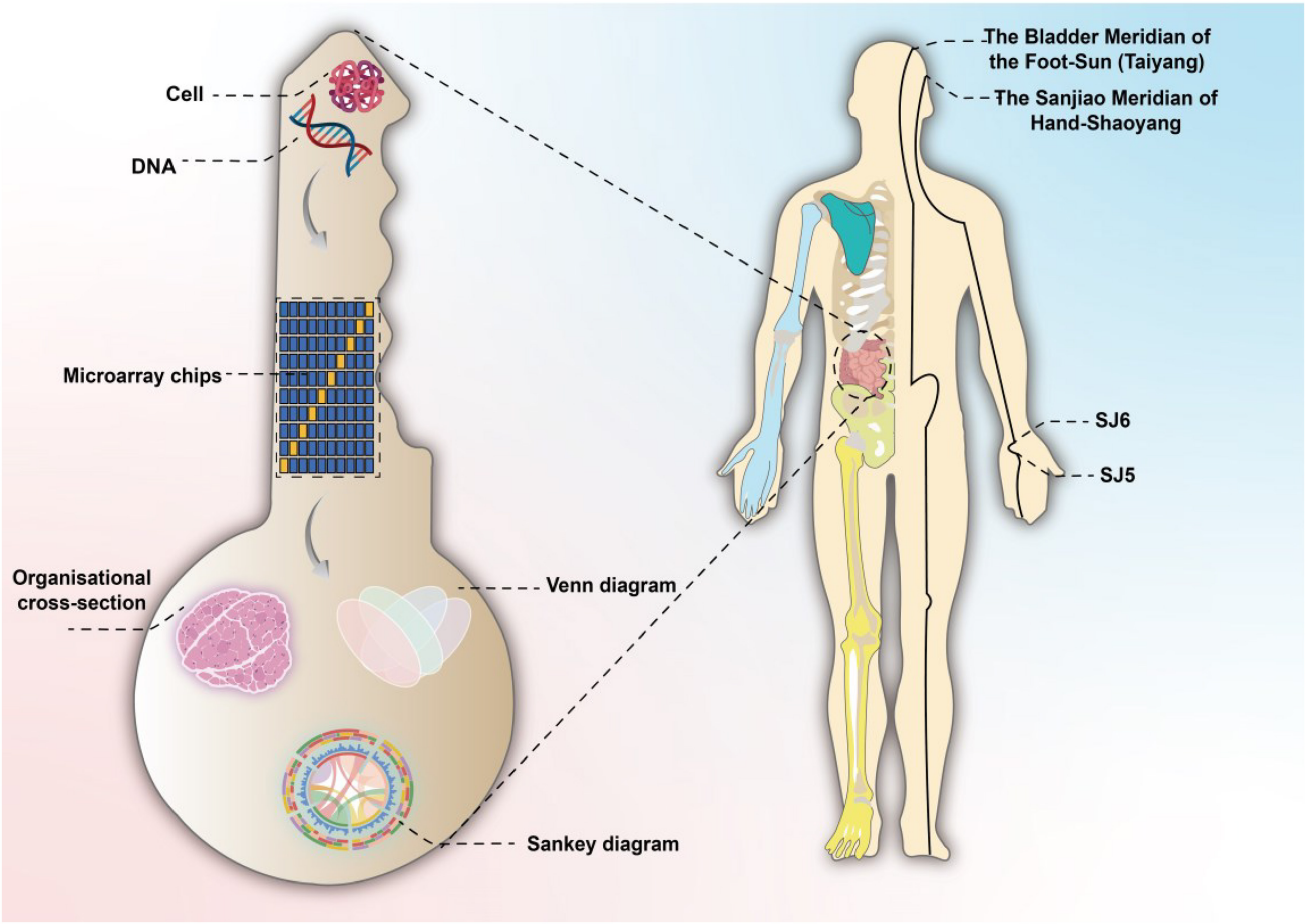
This illustration conceptualizes spatial omics as a transformative key unlocking the complex spatial lock of the human body, represented by meridians and acupoints. The key’s teeth depict core spatial technological principles, including microarray chips for high-throughput spatial barcoding and organizational cross-sections for capturing native tissue architecture. By co-mapping specific cellular components, DNA/RNA molecules, and macro-spatial structures, spatial omics bridges the gap between localized needle stimulation and systemic therapeutic networks (represented by integration tools such as Venn and Sankey diagrams). This provides a powerful, self-explanatory approach to investigate the proposed spatial principles of acupuncture from the micro-cellular microenvironment to the macro-organism network.

The emergence and rapid advancement of spatial omics, a revolutionary technology, holds promise for overcoming this limitation. By integrating genomics, transcriptomics, proteomics, and other omics approaches with spatial information, spatial omics enables the study of biomolecular functions, regulation, and interactions within the native cellular or tissue environment. This provides unprecedented insights into cellular microenvironments, tissue architecture, and disease mechanisms (5). For example, spatial omics techniques can reveal spatiotemporal regulatory patterns of gene expression during tissue and organ formation, as well as the spatial dynamics of cell fate determination (6). In neuroscience, this technology can be used to decipher the cellular composition and gene expression profiles of different brain regions, thereby advancing our understanding of neural circuit functional architecture and the pathological mechanisms of neurodegenerative diseases (7). Collectively, the power of spatial omics to map the molecular mechanisms of tissue architecture and cellular ecosystems makes it an indispensable tool for modern biology, paving the way for its application in elucidating mechanisms of complex interventions like acupuncture (see Figure 1).

This review comprehensively summarizes the spatial and anatomical characteristics of acupoints, their molecular mechanisms of action, the principles of spatial omics technology, and the applications of this technology in both traditional Chinese and Western medicine. We specifically explore the potential of spatial omics to elucidate the spatial basis of acupuncture’s therapeutic effects.

## 2 Spatial and anatomical properties of acupoints

**FIGURE 2:**
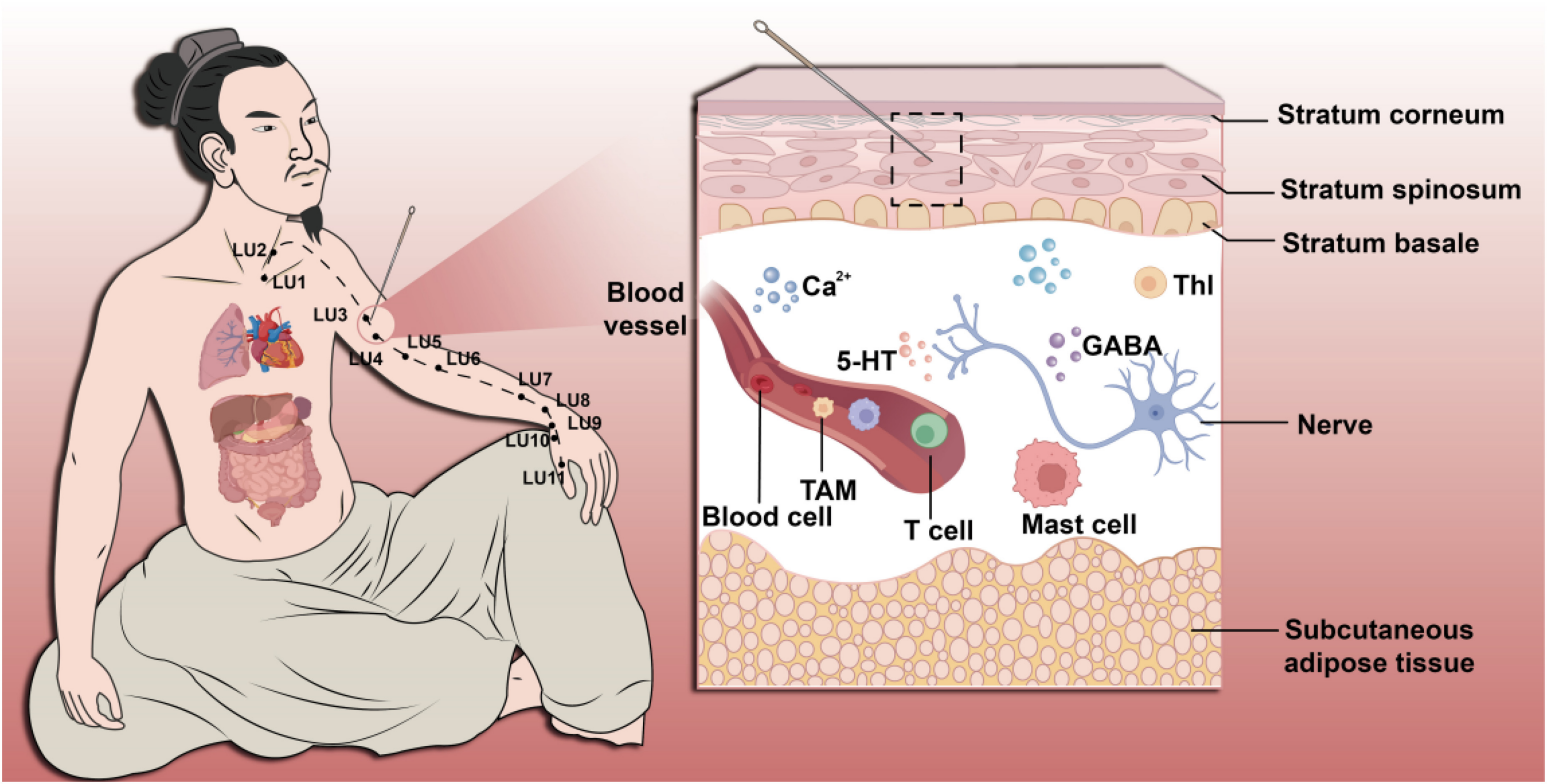
Acupoints are traditionally considered as anatomically specific locations with unique spatial connections to internal organs, where pathological changes are hypothesized to project as localized “disease signals.” This illustration depicts a typical acupoint microenvironment, detailing key spatial structures across stratified anatomical layers: the stratum corneum, stratum spinosum, stratum basale, dermis, and subcutaneous adipose tissue. It highlights the precise spatial distribution of critical cellular components (such as mast cells, T cells, Th1 cells, tumor-associated macrophages TAM, and circulating blood cells) and localized molecular pathways (including Ca^2+^ influx, 5-HT, and GABA neurotransmitter gradients) interacting with nerve endings and microvasculature. Physical stimulation generates biological signals proposed to be spatially transmitted via this neural-connective tissue network, suggesting a conceptual alignment with long-range interstitial flow pathways to modulate systemic therapeutic effects, though direct causal links require further empirical validation.

The therapeutic basis of acupuncture lies in a complex spatial regulatory network that underlies the human body’s functional organization (see Figure 2). First, acupoints are traditionally considered anatomically specific locations on the body surface that maintain unique connections with corresponding internal organs. According to this framework, when an organ enters a pathological physiological state, disease-related signals are thought to project the corresponding acupoint area on the body surface. Therefore, appropriate physical stimulation through needle insertion can exert positive therapeutic effects on the diseased organ (8). Second, meridians are traditionally described as a channel system that circulates “qi” and blood, connects viscera and limbs, and communicates between the exterior and interior, upper and lower parts of the body. This system is believed to regulate systemic functions and integrate the body as an organic whole. When acupoints are stimulated, the resulting effects are hypothesized to influence the flow of “qi” within meridians and its interaction with pathogenic factors. These effects are transmitted and integrated throughout the meridian system, ultimately exerting holistic regulatory effects (9). Guided by this theoretical framework, it is hypothesized that acupoints and meridians may form a spatially interconnected three-dimensional structure, suggesting that the biological signals generated by acupuncture might rely on complex spatial transmission pathways to reach their distant target sites.

Crucially, the clinical efficacy of acupuncture often shows substantial heterogeneity, intricately linked to spatial and anatomical characteristics (10). Variations in therapeutic outcomes are commonly observed across individuals and even with the same acupoint, depending on precise location, needling depth, and manipulation (11). Emerging evidence suggests this heterogeneity arises from micro-spatial biological variations within the acupoint microenvironment. Specifically, the three-dimensional distribution, density, and interactions of local resident cells (e.g., mast cells, fibroblasts), sensory nerve terminals, and microvascular beds directly dictate the initial signal transduction cascade following mechanical stimulation (12). However, conventional bulk tissue analysis requires homogenization, which destroys this spatial architecture and averages out cell-specific signals. Thus, decoding the molecular basis of acupuncture’s clinical heterogeneity and tracing its spatial transmission pathways necessitates mapping the acupoint microenvironment while preserving topological context—a critical bottleneck that calls for spatial omics technologies.

## 3 Spatial networks of acupoint signaling

**FIGURE 3:**
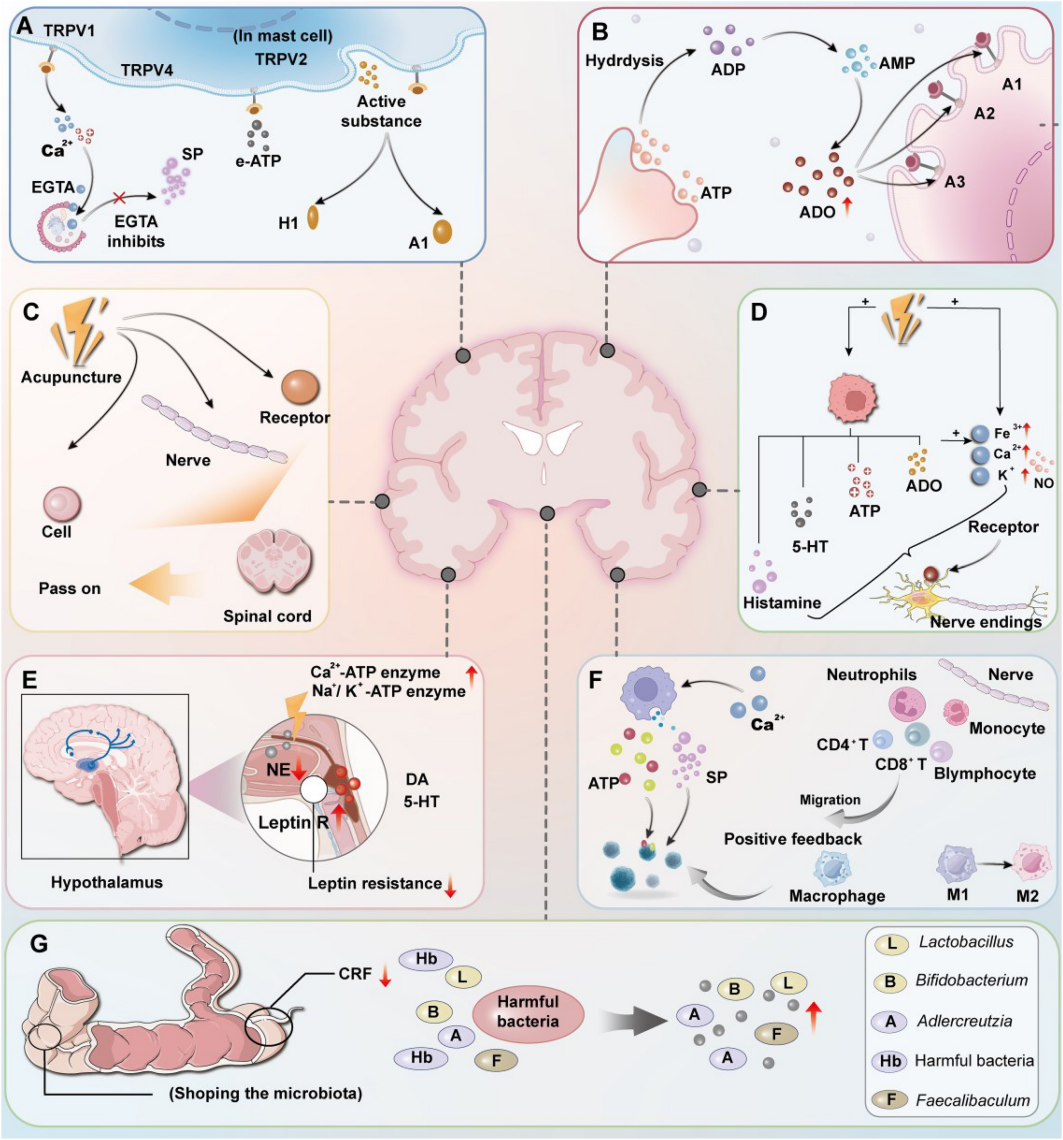
Proposed multi-scale spatial organization mapping the initiation and systemic transmission of acupuncture signals. **(A)** TRPV channels stratify across spatial structures including epidermal (TRPV1), dermal (TRPV2 in mast cells), and interstitial (TRPV4) layers, thought to be involved in mediating thermal, chemical, and mechanical transduction via Ca^2+^ and SP signaling. **(B)** Extracellular ATP hydrolysis establishes a localized purinergic gradient, with adenosine (ADO) acting on A1/A2/A3 receptors to modulate sensory afferents. **(C)** Neural pathways transmit signals from peripheral acupoints to spinal cord and supraspinal centers. **(D)** Cellular components and local chemical mediators (ATP, ADO, 5-HT, NO, Histamine) released from mast cells and nerve endings initiate localized signaling cascades. **(E)** Humoral and endocrine pathways link acupoint stimulation to hypothalamic regulation of neurotransmitters (DA, 5-HT, NE) and metabolic enzymes (Ca^2+^-ATPase, Na+/K+-ATPase). **(F)** Immune responses span local mast cell activation, macrophage polarization (M1/M2), and systemic T/B lymphocyte modulation involving neutrophils and monocytes. **(G)** Acupuncture has been reported to alter gut microbiota composition via corticotropin-releasing factor (CRF) modulation, enriching beneficial genera (*Lactobacillus*, *Bifidobacterium*, *Faecalibaculum*) while reducing harmful bacteria. These spatially organized components are hypothesized to collectively form an integrated network bridging peripheral stimulation to systemic regulation.

The initiation and propagation of acupuncture signals are hypothesized to be governed by the spatial architecture of the acupoint microenvironment and its connections to systemic regulatory networks (see Figure 3). This spatial organization—encompassing the layered distribution of molecular sensors, the compartmentalized arrangement of neural and immune elements, and the formation of local chemical gradients—is thought to determine how mechanical stimulation at peripheral sites is transduced into region-specific and system-level effects.

### 3.1 Spatial organization of molecular sensors in the acupoint microenvironment

#### 3.1.1 TRPV channels stratified distribution across tissue layers

Transient receptor potential vanilloid (TRPV) channels serve as primary transducers of acupuncture-related mechanical and thermal stimuli, displaying characteristic spatial segregation across distinct tissue layers within the acupoint microenvironment (13, 14) (see Figure 3A). Within the epidermis, TRPV1 is predominantly expressed in keratinocytes and dermal sensory nerve endings, where it primarily mediates thermal and chemical stimulus perception (15–17). This localization underlies the mechanism of moxibustion: elevated skin temperature activates TRPV1 channels, promoting Ca^2+^ influx and triggering downstream events such as substance P (SP) release and neuropeptide synthesis, ultimately exerting anti-inflammatory and analgesic effects (13, 18–20). Consistent with this spatial model, chelation of extracellular Ca^2+^ with EGTA attenuates these effects, confirming that local Ca^2+^ influx at the epidermal level is critical for moxibustion efficacy (18).

In the dermis, TRPV2 localizes to mast cells (MCs), where acupuncture stimulation activates these channels and triggers MC degranulation (21). The released bioactive substances, including histamine and adenosine, act on H1 and A1 receptors at adjacent sensory nerve endings, converting mechanical input into nerve impulses that propagate upward (22, 23). This dermal compartment thus serves as a key site for translating mechanical force into chemical signals.

Within the interstitium, TRPV4 exhibits high sensitivity to mechanical stress and is enriched in fibroblasts and sensory nerve terminals (24, 25). Experimental blockade of TRPV4 at the ST36 acupoint completely abolishes the anti-nociceptive effects of acupuncture, underscoring its essential role in mechanotransduction (26). Simultaneous TRPV4 activation enhances ecto-ATPase activity, leading to transient accumulation of extracellular ATP critical initiating signal that activates downstream purinergic pathways and ultimately mediates acupuncture analgesia (26).

Collectively, this stratified distribution—epidermal TRPV1 for thermosensation, dermal mast cell-associated TRPV2 for chemical signal generation, and interstitial TRPV4 for mechanotransduction—reveals how the spatial organization of TRPV channels within the acupoint microenvironment enables the integration of diverse acupuncture stimuli into a coordinated signaling output.

#### 3.1.2 Purinergic signaling gradients from ATP release to adenosine reception

Acupuncture stimulation at specific points triggers local ATP release from multiple cell types, including MCs, fibroblasts, and sensory nerves. Once released, ATP undergoes sequential hydrolysis via a cascade of extracellular nucleotidases, generating adenosine diphosphate, adenosine monophosphate, and ultimately adenosine (27) (see Figure 3B). This enzymatic cascade establishes a spatiotemporal concentration gradient of purinergic metabolites within the interstitial space, with adenosine levels highest at the needle site and diminishing radially.

Adenosine serves as a key effector molecule in this signaling axis, binding to A1, A2, and A3 receptors on adjacent sensory nerve endings to exert anti-nociceptive effects (28–30). The functional importance of this spatial arrangement is supported by multiple lines of evidence. Electroacupuncture (EA) elevates local adenosine levels, activates adenosine receptor signaling, and subsequently inhibits SP release from dorsal root ganglia, downregulates NK-1R expression, and reduces inflammatory mediators such as CD68 and TNF-α, thereby decreasing peripheral nociceptor excitability (31). Direct validation comes from pharmacological experiments: injection of an A1 receptor agonist at the ST36 acupoint mimics the analgesic effects of EA, whereas disruption of afferent signaling via dorsal nerve root rhizotomy or local administration of the SP receptor antagonist CP96345 abolishes EA-induced analgesia, accompanied by rebound expression of downstream inflammatory molecules (32).

Collectively, these findings indicate that the spatial organization of purinergic signaling may serve as one of the key mechanisms underlying the local effects of acupuncture, offering a conceptual framework for decoding the biological encoding of acupuncture intensity. This gradient-based mechanism operates primarily at the site of stimulation, complementing the neural, endocrine, and immune pathways that mediate the remote therapeutic actions of acupuncture.

### 3.2 Cellular neighborhoods and intercellular communication at acupoints

#### 3.2.1 Neural trajectories and acupoint compartments

The spatial organization of neural structures within and around acupoints determines how local stimulation is transformed into ascending signals that reach the central nervous system. Anatomical studies have revealed consistent associations between classical meridian distributions and specific peripheral nerves. The Lung Meridian correlates with the median nerve, the Pericardium Meridian with the territories of the radial and ulnar nerves, and the Bladder and Stomach Meridians with the pudendal nerve (33). These topographic alignments indicate that acupoints are not randomly distributed but rather positioned at sites where needles can engage nerve endings, peripheral nerve trunks, or their branches.

Upon needle insertion, sensory receptors in the skin and muscles are activated, transmitting information to primary and secondary neurons in the spinal cord and subsequently to higher centers (34) (see Figure 3C). This neural trajectory, from peripheral nerve terminals to spinal segments and supraspinal structures, constitutes an anatomically defined pathway that facilitates the propagation of acupuncture signals. The functional relevance of this spatial architecture is supported by observations that neuropathic changes at any level of this pathway, whether local, spinal, or supraspinal, typically diminish or abolish the therapeutic effects of acupuncture, indicating that intact neural connections are necessary for efficacy.

This anatomical compartmentalization suggests that local neural activation at acupoints may represent one of the structural starting points for the systemic effects of acupuncture, although the precise mechanisms by which local stimulation translates into remote regulatory effects remain to be fully elucidated.

#### 3.2.2 Local tissue reactions and chemical signals

The local tissue environment at acupoints exhibits distinctive structural and chemical features that contribute to signal initiation. Compared with adjacent non-acupoint tissues, most acupoints display enriched neural structures, hair follicles, sweat glands, and vascular networks (35–38). This specialized architecture provides an anatomical substrate for generating local chemical signals upon acupuncture stimulation, which then act on sensory afferents to initiate downstream signaling cascades.

Mast cells represent one important source of local chemical signals. When acupuncture manipulates the skin’s collagen network, MCs are activated—directly or indirectly—and release multiple mediators into the interstitial space, including histamine, serotonin, ATP, and adenosine (see Figure 3D). These mediators subsequently activate corresponding receptors on the terminals of peripheral sensory ganglion neurons. Experimental evidence supports this model: acupuncture at ST36 in healthy volunteers significantly increased adenosine concentration in the tissue interstitium (39); stimulation at LI4 in rats promoted mast cell degranulation and histamine release (40); and acupuncture at ST36 in arthritic mice increased local accumulation of ATP, ADP, AMP, and adenosine, an effect that was abolished by pharmacological inhibition of mast cell degranulation with sodium cromoglycate, accompanied by a reduction in analgesic efficacy (41).

Nitric oxide (NO) and SP constitute additional chemical signals enriched at acupoints. Studies indicate that both NO synthase (NOS) protein expression and NO concentrations in skin tissues located within acupoint regions are significantly higher than in non-acupoint areas; acupuncture further elevates NOS activity and NO content in these regions (38, 42). Evans blue staining has revealed that acupuncture promotes local SP release at acupoints, which enhances sensory afferent sensitivity to needle stimulation and facilitates signal transduction, with functional relevance demonstrated in hypertension models (43).

Acupuncture intervention also alters the ionic composition of the acupoint microenvironment. Stimulation significantly elevates local concentrations of Ca^2+^, K^+^, Na^+^, and Cl^–^ in acupoint regions (44, 45), with Ca^2+^ increases observed around acupoints and along specific subcutaneous connective tissue pathways (46). This Ca^2+^ elevation promotes mast cell degranulation (47); conversely, MCs degranulation may further contribute to local Ca^2+^ mobilization, establishing a potential positive feedback loop. Functional studies show that subcutaneous Ca^2+^ infusion mimics the therapeutic effects of acupuncture, whereas Ca^2+^ channel blockade or antagonism of Ca^2+^-binding proteins partially attenuates its efficacy. Beyond these physiologically relevant ions, metal element analysis has identified Fe^3+^ as the most abundant metal at acupoints (48). These observations raise the possibility of a distinctive redox microenvironment at acupoint sites, although the physiological significance of this finding remains to be further investigated (48, 49).

Together, these findings illustrate that the acupoint microenvironment contains a diverse array of chemical signals, derived from MCs, vascular and neural elements, and ionic components, that collectively contribute to the initiation and local modulation of acupuncture responses.

#### 3.2.3 Body fluids and endocrine signals

Beyond local chemical signals, acupuncture exerts systemic effects through the modulation of humoral and endocrine pathways. These remote regulatory actions involve alterations in circulating hormone levels, neuroendocrine signaling, and metabolic mediators that collectively contribute to therapeutic outcomes across diverse pathological conditions.

Animal studies have provided mechanistic insights into these remote effects. In a chronic restraint stress-induced depression mouse model, seven sessions of acupuncture at four acupoints (KI10, LR8, LU8, LR4) significantly reduced plasma leptin levels while increasing leptin receptor expression in the hypothalamus and hippocampus, suggesting improved leptin sensitivity (50). In a high-fat diet-induced obesity rat model, EA at lower limb points (ST36, ST44) or abdominal points (ST25) reduced serum levels of leptin, resistin, and TNF-α, while elevating adiponectin and cholecystokinin-8; this intervention also altered the discharge activity of glucose-inhibitory neurons in the lateral hypothalamic area, providing a potential mechanistic link to feeding behavior regulation (51). Additionally, in obese rats, bilateral acupuncture at ST36 and ST44 significantly downregulated elevated norepinephrine levels in the lateral hypothalamic area while restoring serotonin, dopamine, and the activity of Na^+^/K^+^-ATPase and Ca^2+^-ATPase in this region (52) (see Figure 3E). These observations indicate that acupuncture can modulate central neurotransmitter systems and energy metabolism at the hypothalamic level, although whether these changes represent correction of pre-existing dysregulation or broader physiological responses remains to be determined.

Clinical observations have extended these mechanistic findings to patient populations, albeit with inherent limitations. A study involving 30 gastrointestinal cancer patients with cachexia reported that eight sessions of acupuncture significantly reduced blood lactate dehydrogenase and serum leptin concentrations while lowering ghrelin levels (53). While these changes are consistent with modulation of the leptin-ghrelin axis, the small sample size and absence of a control group limit causal inference regarding metabolic-inflammatory effects.

Together, these findings indicate that acupuncture can engage systemic humoral and endocrine pathways extending beyond the local acupoint microenvironment. The observed alterations in hypothalamic neurotransmitter systems, hypothalamic neuronal activity, and circulating metabolic hormones suggest the involvement of neuroendocrine axes as key directions for future mechanistic investigation. Elucidating how local acupoint stimulation engages these remote pathways will be essential for establishing a unified framework of acupuncture action.

#### 3.2.4 Immunoregulatory signals

Acupuncture engages immune responses at multiple spatial scales—from local activation of resident immune cells within the acupoint microenvironment to systemic modulation of circulating immune populations and remote neuro-immune interfaces (see Figure 3F).

Local immune activation at acupoints begins with the spatial organization of resident immune cells. Studies reveal that mast cell density in the epidermis and dermis of acupoint regions is significantly higher than in non-acupoint areas (54, 55). Moreover, mast cell aggregation is observable near small blood vessels, nerve bundles, and their terminals along specific subcutaneous connective tissue pathways (56). The mechanical stress generated by needle insertion and twisting stimulates mechanosensitive channels on mast cell surfaces (e.g., TRPV2) and stretch-activated chloride channels. This mechanical input induces Ca^2+^ influx, which promotes mast cell degranulation and the release of bioactive substances including ATP, SP, and histamine (23, 40, 57–60). These released mediators subsequently activate corresponding receptors, such as adenosine A1 and histamine H1 receptors, on adjacent sensory nerve terminals (23, 57). The released mediators diffuse into the extracellular space, where they may further promote MCs activation, contributing to local signal amplification (61). In this context, MCs represent one important cellular hub for initiating the neuroimmune cascade at acupoints, alongside other resident cells such as Langerhans cells and macrophages (62, 63).

Systemic immunomodulatory effects have been documented across multiple experimental and clinical contexts. In human subjects, acupuncture treatment was associated with increased peripheral blood populations of CD3^+^, CD4^+^, CD8^+^, CD11b^+^, CD16^+^, and CD56^+^ cells, accompanied by elevated serum levels of various cytokines (64). In allergic asthma patients, acupuncture reduced serum total IgE concentration and promoted proliferation of peripheral blood CD3^+^, CD4^+^, and CD8^+^ T lymphocytes (65). In a collagen-induced arthritis mouse model, acupuncture was associated with normalization of the CD4^+^/CD8^+^ T ratio and suppression of inflammatory responses (66).

Mechanistic studies have identified specific neuro-immune pathways underlying these remote effects. Landmark studies demonstrated that acupuncture stimulation at ST36 activates splenic sympathetic nerves, engaging the β2-adrenergic receptor-cAMP pathway in B cells and CD4^+^ T cells and promoting their differentiation toward an anti-inflammatory phenotype; this effect was attenuated following pharmacological blockade of splenic sympathetic nerves with 6-hydroxydopamine (67). EA stimulation has also been shown to modulate alveolar macrophage polarization, inhibiting differentiation toward the pro-inflammatory M1 phenotype and reducing local pro-inflammatory mediator secretion (68).

Collectively, these findings indicate that acupuncture engages immune responses across multiple spatial compartments—from locally enriched MCs at acupoints to remote neuro-immune interfaces involving the sympathetic innervation of lymphoid organs. While the precise mechanisms linking local acupoint stimulation to these systemic immunomodulatory effects continue to be investigated, the emerging picture points to an integrated neuro-immune axis that bridges peripheral stimulation with central and peripheral immune regulation.

#### 3.2.5 Gut microbiota

Acupuncture has been associated with alterations in gut microbiota composition, a distal effect that reflects the broad physiological influence of acupoint stimulation. In a model of irritable bowel syndrome (IBS), EA at bilateral ST25 and ST36 significantly suppressed colonic corticotropin-releasing factor overexpression, enhanced microbial α-diversity, and increased the relative abundance of *Lactobacillus* and *Bifidobacterium*—genera commonly regarded as beneficial in this context (see Figure 3G). These changes were accompanied by reduced visceral hypersensitivity induced by colonic distension (69).

In obese rat models, eight weeks of EA at GB26, CV12, ST36, and ST40 was associated with improved blood glucose, lipid profiles, and hepatic steatosis, alongside alterations in α-diversity and the Firmicutes/Bacteroidetes ratio. This intervention also increased the relative abundance of bacteria with predicted capacity for short-chain fatty acid production, including Adlercreutzia and Faecalibaculum (70). While these studies demonstrate correlative associations between acupuncture and gut microbiota composition, the causal contribution of microbial changes to observed phenotypic improvements remains unestablished. Integrating spatial omics with microbial profiling and functional validation may ultimately clarify how peripheral stimulation translates into distal microbial and physiological remodeling.

## 4 Spatial omics technologies and applications

**FIGURE 4:**
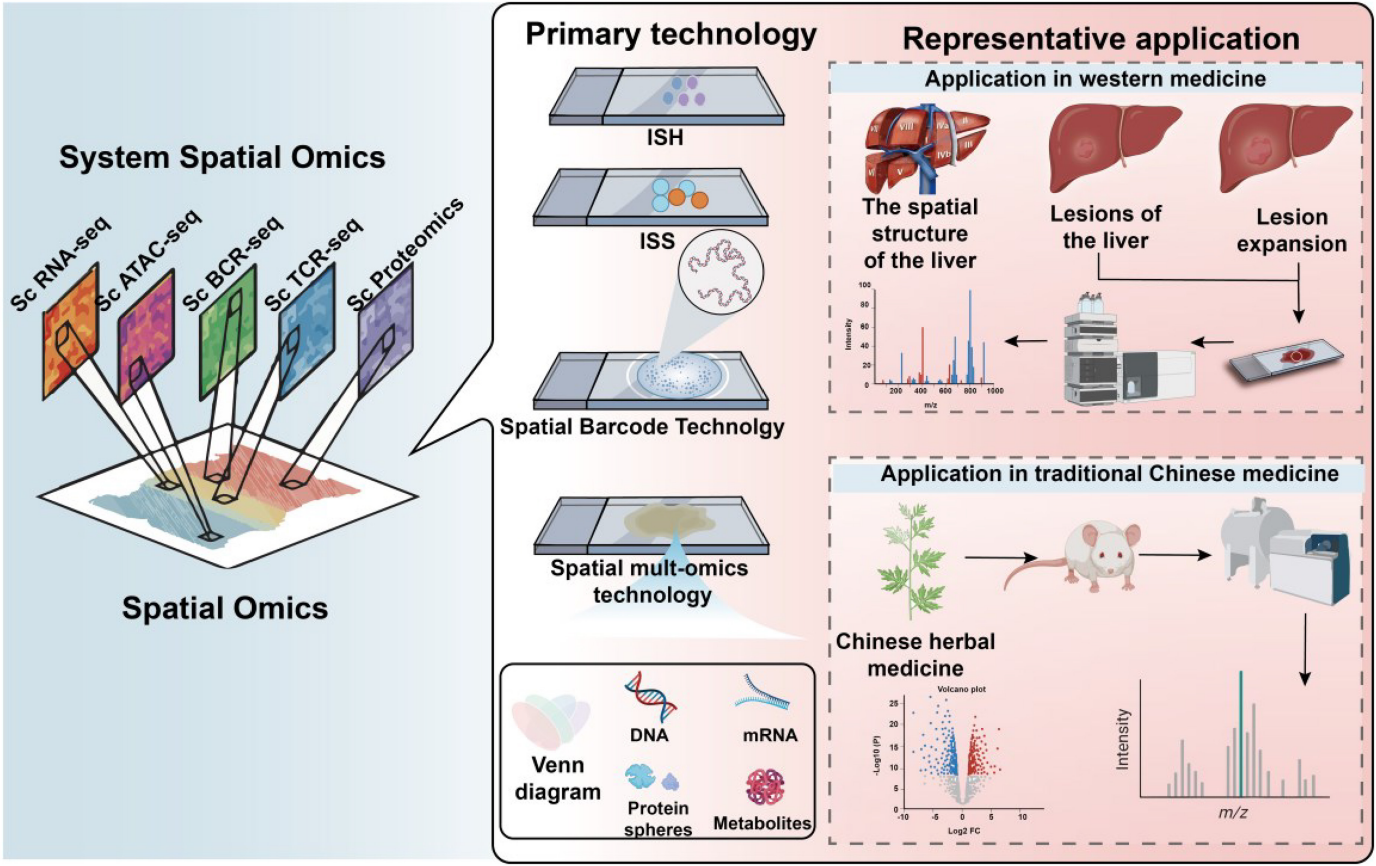
Spatial omics bridges molecular biology and macroscopic tissue anatomy by preserving the exact spatial context of cellular data. The technological framework is built upon three core methodologies: *in situ* hybridization (ISH) for targeted RNA imaging, *in situ* sequencing (ISS) for direct spatial sequencing, and high-resolution Spatial Barcode Technology for capturing genome-wide transcripts. These methodologies converge into integrated spatial multi-omics platforms, enabling the simultaneous co-mapping of diverse molecular layers (DNA, mRNA, proteins, and metabolites) within a spatial context, as conceptually represented by a Venn diagram. In Western medicine, this approach is revolutionizing our understanding of tissue spatial architecture (e.g., functional zonation of the liver) and the spatial dynamics of lesion expansion. In TCM, it offers potential analytical tools for the quality evaluation of Chinese herbal medicines (e.g., via mass spectrometry imaging) and may help to investigate the spatial pharmacological mechanisms of complex formulae in *in vivo* models.

The essence of multi-cellular life activities is highly spatialized and organized. The spatial order in which cells form tissues and organs determines an organism’s function, development, homeostasis, and the onset and progression of disease. For example, precise control of spatial information is crucial for embryonic development; cells must know their coordinates within the embryo to differentiate correctly and migrate to the right locations to form organs. Disease onset often exhibits spatial characteristics, such as neurodegeneration initiating at specific sites or cancer tissues possessing distinct tumor microenvironments. While traditional omics technologies provide high-resolution molecular maps, they require dissociating tissues into individual cells, thereby losing spatial positioning information within the original tissue. Metaphorically, this is akin to reconstructing an unknown vase from tiny fragments, making accurate results difficult to guarantee. Fortunately, emerging spatial omics technologies can simultaneously preserve the original spatial positioning of tissue while acquiring molecular information at the cellular or subcellular level in a high-throughput manner (see Figure 4). This bridges the gap between microscopic molecular biology and macroscopic tissue pathology, opening entirely new dimensions for understanding developmental, physiological, and disease mechanisms (71–73). To facilitate a clearer methodological foundation for acupuncture research, the technical classification, core specifications, inherent trade-offs, and applicable scenarios of these spatial omics platforms are systematically summarized and cross-compared in Table 1.

**TABLE 1:** Methodological comparison, technical trade-offs, and applicable acupuncture research scenarios of major spatial omics platforms.

Technology category	*In situ* hybridization (ISH-based)	*In situ* sequencing (ISS-based)	Spatial barcoding (next-gene sequencing)	Multi-omics integration platforms
Representative platforms	seqFISH, MERFISH	FISSEQ, STARmap (74)	10x Genomics Visium (74), stereo-seq (74–76)	DBiT-seq (74), imaging mass cytometry (77)
Spatial resolution	Sub-cellular (< 100 nm to 1 μm)	Sub-cellular (∼200 nm to 1 μm)	Spot-level to Sub-cellular (55 μm down to 500 nm)	Modality-dependent (from 1 μm to 55 μm)
Throughput (genes/targets)	Medium to high (hundreds to thousands)	Medium to high (hundreds to thousands)	Genome-wide (Unbiased, >20,000 genes)	Multi-modal (simultaneous RNA and tens of proteins)
Key strengths/advantages	High detection efficiency and sensitivity; preserves accurate subcellular transcripts.	Reads sequences directly in situ; high spatial precision; low false-positive rates.	Unbiased, genome-wide profiling; high sample throughput; compatible with standard frozen/FFPE tissues.	Captures direct correlations between RNA expression and functional protein translation *in situ*.
Inherent limitations	Prone to optical crowding; complex probe design and lengthy imaging cycles; requires custom fluidics.	Relies on enzymatic reactions; lower detection efficiency for low-abundance transcripts compared to ISH.	Resolution can be limited by spot pitch (e.g., visium); RNA capture efficiency decreases at ultra-high resolution.	Highly complex computational workflows for data alignment; expensive reagents; trade-offs in multi-modal sensitivity.
Applicable scenarios in acupuncture	Mapping precise sub-cellular molecular architecture and receptor localizations within acupoint microenvironments.	Identifying specific cell types and spatial variations in signaling molecules directly within intact skin/muscle layers of acupoints.	Screening for novel biomarkers and untargeted, global transcriptomic alterations across long-distance interstitial flow pathways.	Interrogating the integrated multi-scale regulatory networks (e.g., cytokine translation vs. transcript shifts) during acupuncture.

ISH, *in situ* hybridization; ISS, *in situ* sequencing; NGS, next-generation sequencing; FFPE, formalin-fixed paraffin-embedded; RNA, ribonucleic acid; DNA, deoxyribonucleic acid; μm, micrometer; nm, nanometer; targets, genes or molecular features. *In situ* is presented in italics in accordance with biological nomenclature guidelines.

### 4.1 Spatial omics technologies

#### 4.1.1 *In situ* hybridization techniques

*In situ* hybridization is the most widely applied technique in spatial genomics, analyzing spatial information by detecting the expression of specific genes in tissue sections (78). Early single-molecule fluorescence *in situ* hybridization (smFISH) relied on fluorescently labeled single-stranded oligonucleotide probes that specifically bind to target RNA molecules. Fluorescence signals emitted by these probes are captured via fluorescence microscopy, enabling quantitative analysis of RNA abundance and localization (79–81). While smFISH offers single-molecule resolution, it is limited in the number of genes it can detect. To overcome this constraint, multiplex error-robust fluorescence *in situ* hybridization (MERFISH) was developed. MERFISH employs multiplex hybridization imaging and combinatorial labeling strategies to simultaneously detect thousands of RNA molecules. This technique utilizes error-robust barcoding and serialized imaging steps, significantly enhancing detection throughput and sensitivity (82–85). Furthermore, sequence fluorescent *in situ* hybridization (seqFISH^+^) technology employs sequential hybridization steps to further expand the number of detectable genes, enabling simultaneous detection of over 10,000 genes. seqFISH^+^ also utilizes pseudo-color channels and optimized imaging strategies to further enhance detection efficiency and resolution (6).

#### 4.1.2 *In situ* sequencing technology

*In situ* sequencing (ISS) is another crucial technique in the field of spatial genomics, enabling simultaneous detection of multiple genes by reading target nucleic acid sequences directly within tissue sections (86). It generates clonal rolling circle products via rolling circle amplification, followed by sequencing to read target sequences (6). However, first-generation ISS technologies exhibited low efficiency in in situ reverse transcription to form cDNA, resulting in overall poor detection performance. The recently developed STARmap approach bypasses the reverse transcription step by using dual primers to form DNA nanospheres, significantly enhancing detection efficiency. Furthermore, STARmap optimizes hydrogel histochemistry and integrates a novel dual-base sequencing strategy to allow for the detection of thousands of genes (87).

*In situ* sequencing technologies not only provide targeted detection, but also facilitate non-targeted whole-transcriptome analysis. For example, by using random hexamers equipped with adapter sequences, Fluorescent in situ RNA sequencing performs non-targeted cDNA synthesis with the potential for comprehensive transcriptome coverage. Combined with expanded sequencing technology, which leverages advances in extended microscopy, spatial resolution and detection efficiency in ISS can be further enhanced (88).

#### 4.1.3 Spatial barcoding technology

Spatial barcoding technology is an emerging spatial detection technique that relies on barcodes carrying spatial location information to capture probes for high-throughput transcriptomic analysis of molecular information in tissue sections (89). Spatial transcriptomics (Visium) is one of the earliest commercialized spatial barcoding technologies. It utilizes microarray printing to print barcodes with known sequences and polyT probes at specific intervals onto glass surfaces, forming capture carriers. When mRNA from tissue sections contacts these carriers, in situ reverse transcription synthesizes cDNA. High-throughput sequencing then yields transcriptomic data with spatial positioning information (90). However, Visium is constrained by its physical printing limitations, with a 100 μm spacing between capture points resulting in relatively low spatial resolution. In contrast, Slide-seq employs magnetic beads with a diameter of 10 μm, each bearing a unique barcode, enabling 10-micron resolution (89). A technology that takes this further is high-density spatial transcriptomics, which uses a randomly ordered beads to achieve a superior resolution of 2 μm (91). Through RCA-based amplification of DNA nanospheres from random barcodes, Stereo-seq creates a platform that achieves panoramic, single-experiment analysis across subcellular to organ scales (92, 93).

#### 4.1.4 Spatial multi-omics integration technology

Spatial multi-omics integration technology enables simultaneous analysis of multiple molecular information (including gene expression, proteins, metabolites, epigenetic marks, etc.) within biological tissues while preserving molecular spatial positioning (94). Spatial CITE-seq technology employs antibodies labeled with DNA barcodes to concurrently detect transcriptomic and proteomic data. This technique achieves quantitative protein analysis by binding antibodies to target proteins and subsequently detecting the DNA barcodes on the antibodies via sequencing (95). Spatial ATAC-seq combines chromatin accessibility sequencing (ATAC-seq) with transcriptomics can identify open chromatin regions and reveal the epigenetic mechanisms governing gene regulation (96). Furthermore, spatial CUT&Tag technology integrates chromatin immunoprecipitation with transcriptomics to investigate the relationship between histone modifications and gene expression (6, 96). These multi-omics integration technologies not only provide richer cellular and tissue information but also offer crucial tools for investigating gene expression regulation mechanisms and cellular functions, thereby providing new perspectives and depth for biological and medical research.

### 4.2 Advances in spatial omics applications

#### 4.2.1 Applications in western medicine

##### 4.2.1.1 Spatial architecture analysis of biological systems

The spatial architecture of biological systems refers to the spatial distribution and interrelationships of various molecules, cells, and tissues within an organism. These specific spatial arrangements profoundly influence the functional execution of biomolecules and are crucial for the precise operation of the entire biological system. In recent years, by integrating multiple single-cell sequencing technologies with spatial transcriptomics approaches, researchers have achieved significant breakthroughs in deciphering the spatial architecture of biological systems. Fang et al utilized multiplex error-robust fluorescence *in situ* hybridization (MERFISH) to perform single-cell transcriptomic imaging of the human middle temporal gyrus and superior temporal gyrus. They discovered that excitatory and inhibitory neurons exhibit distinct layered distributions across cortical depths, while non-neuronal cells (such as oligodendrocytes) are enriched in deeper layers and white matter (96). Parallel findings show that L6b neurons are widely dispersed throughout the human cortex, whereas they form a thin layer in mice, indicating significant differences in the spatial architecture of the brain between species (97). These findings fully demonstrate the immense application potential of spatial omics technologies in deciphering the three-dimensional structures of biological systems.

##### 4.2.1.2 The spatial science basis for disease onset and progression

Disease represents both a disruption of biochemical pathways and a collapse of spatial order. Deeply analyzing the spatial characteristics of disease formation and progression holds promise for driving innovation in therapeutic strategies. Kuppe et al. integrated multi-modal omics data—including single-cell gene expression, chromatin accessibility, and spatial transcriptomics—revealing distinct stress states in cardiomyocytes within the infarct core versus border zone. Additionally, fibroblasts exhibited marked spatial clustering during fibrosis (98). Sun et al. employed mass spectrometry imaging -based spatial metabolomics and lipidomics, combined with microarray-based spatial transcriptomics, to conduct a hierarchical visualization study of metabolic heterogeneity and cellular metabolic interactions in gastric cancer samples. They identified an immune- and inflammation-associated zone rich in plasma cells, follicular B cells, and Th2-like CD4^+^ T cells at the tumor-adjacent tissue interface (99). This zone could be subdivided into peritumoral lymphoid tissue (PLT) and distal lymphoid tissue with distinct metabolic signatures (99); where the PLT region exhibited marked metabolic reprogramming, including enhanced glutamine metabolism and upregulation of fatty acid synthase and inflammatory mediator synthase expression, suggesting a potentially stronger antitumor immune response in this area (99).

Similarly, Ravi et al. (100) integrated spatial transcriptomics, metabolomics, and proteomics data to reveal that reactive immune zones (highly infiltrated with immune cells) in glioma tissues significantly enriched memory T cells, exhausted T cells, and CD163^+^ myeloid cells, exhibiting immune-suppressive features characterized by high PD-1 expression. Analysis of mouse colon tissue at day 14 post-DSS induction by Annika et al. (101) revealed that B cells predominantly clustered in the injured region and showed high correlation with genes associated with tissue repair. By integrating single-cell RNA sequencing data with spatial transcriptomics, they further revealed that B cell presence significantly reduced interactions between intestinal epithelial cells (IECs) and stromal cells; conversely, these interactions markedly increased in B cell-depleted mice. Moreover, following B cell depletion, the spatial distance between stromal cells (e.g., Bmp5^+^ stromal cells) and IECs (e.g., E1 and EMT cells) markedly decreased, indicating that B cell depletion facilitates the restoration of normal epithelial-stromal cell interactions.

##### 4.2.1.3 Spatio-temporal evolution mechanism

Biological processes represent a meticulously orchestrated symphony of precise coordination across temporal and spatial dimensions. Individual development, homeostasis maintenance, disease formation, and species evolution are all intricate processes intertwined and synergistically interacting across temporal and spatial dimensions. Arutyunyan et al. combined spatial transcriptomics with single-cell transcriptomics to map a spatially resolved single-cell atlas of early pregnancy trophoblast cell (EVT) development, and revealed that EVT cells invade the uterine decidua from the placenta to form cellular columns, subsequently differentiating into invasive EVT and endovascular EVT (102).

As a fundamental physiological process, wound healing represents another quintessential example of such spatiotemporal coordination and its dysregulation. Liu et al. (103) performed single-cell multi-omics analysis on skin wounds from the same individual across inflammatory, proliferative, and remodeling phases, resolving cellular and molecular dynamics during healing with high spatiotemporal resolution. They defined the precise cellular architecture at the wound margin and identified FOSL1 as a key driver of re-epithelialization. This spatial perspective further uncovered a relay mechanism, revealing how pro-inflammatory macrophages and fibroblasts sequentially interact across different zones to guide keratinocyte migration. Moreover, comparison with chronic wounds showed a spatial dissociation between keratinocytes and immune signals, linking healing failure to a breakdown in coordinated cellular positioning.

#### 4.2.2 Applications in TCM

##### 4.2.2.1 Evaluation of medicinal herb quality

The evaluation of Chinese herbal medicine quality serves as a crucial foundation for ensuring the safety and efficacy of TCM. Spatially resolved analysis technologies, such as mass spectrometry imaging, enable *in situ* analysis of herbal components, proteins, and genes, addressing the limitations of traditional analytical methods that lose spatial information. This approach provides more comprehensive, accurate, and rich biological insights for herbal quality assessment. For example, Yu et al. utilized matrix-assisted laser desorption/ionization time-of-flight mass spectrometry imaging (MALDI-TOF MS) to analyze the spatial distribution of metabolites within nodular structures (termed “nail heads”) present near the apical regions of Panax notoginseng roots, lateral roots, and main roots. Results revealed that nail heads originate from undeveloped lateral roots, and their number correlates positively with ginsenoside R1 content, suggesting nail heads serve as a key indicator of Panax notoginseng quality (104). Similarly, Sun et al. employed matrix-assisted laser desorption/ionization time-of-flight mass spectrometry imaging to reveal that notoginsenoside-R1 predominantly distributed in the phloem and outer xylem of Panax notoginseng, whereas ginsenoside-Re exhibited higher concentrations in the pith and inner xylem (105). Guo et al. used air-assisted desorption/ionization time-of-flight mass spectrometry imaging to visualize metabolite distribution in Pueraria root with high spatial resolution. Results indicated that carbohydrates, vitamins, and inosine monophosphate were enriched in the Phloem of *Pueraria thomsonii Benth* (*P. thomsonii*) and the xylem of *Pueraria lobata* (willd.) Ohwi (*P. lobata*), respectively; whereas 3-hydroxypuerarin exhibited an inverse distribution pattern (106).

##### 4.2.2.2 Pharmacological mechanism research

Spatial omics technologies also offer novel perspectives for elucidating the pharmacology of TCM. Li et al. (107) investigated the protective mechanism of the Double Ginseng Cardio-Protective Formula against myocardial ischemia-reperfusion injury using MALDI-TOF MS and found that it significantly increased AMP, ATP, and GMP levels in the ischemic infarction zone, reduced energy charge in myocardial tissue, and markedly elevated levels of membrane phospholipids such as PC (22:0), PC (38:4), and PC (36:1). Zhou et al. employed spatial transcriptomics and single-cell analysis to reveal that high-fat diet-induced NAFLD in mice was characterized by a significant reduction in hepatocyte numbers, accompanied by substantial accumulation of associated neutrophils, macrophages, monocytes, B cells, and T cells around hepatic endothelium. However, Jiangtang Qingre Formula treatment markedly reversed these trends and improved inflammatory and fibrotic severity in mouse livers (108). By integrating spatial metabolomics and network pharmacology, Fan et al. (109) identified that the ginseng-schisandra herb pair acts against Alzheimer’s disease by modulating neurotransmitters (glutamate, GABA) and enhancing TCA cycle metabolites (citrate, fumarate), thus alleviating energy metabolism disorders. The treatment also significantly influenced the metabolism of polyunsaturated fatty acids and phospholipids (e.g., phosphatidylcholine, phosphatidylethanolamine), which in turn exerted neuroprotective effects.

## 5 Spatial omics in action: future directions for acupuncture research

Building on the technological capabilities outlined above, this section proposes a forward-looking framework for applying spatial omics to key unresolved questions in acupuncture research. We outline three potential avenues of investigation: first, decoding the spatial molecular architecture of acupoints; second, capturing the spatiotemporal dynamics of local signal initiation; and third, mapping remote network effects through multi-organ spatial mapping. Subsequently, we candidly discuss the current limitations, open controversies, and empirical gaps that must be addressed to transform these conceptual directions into testable hypotheses.

### 5.1 Mapping the spatial architecture of acupoints: from descriptive morphology to molecular cartography

The structure of acupoints is not a single, specific entity, but rather a region where multiple tissue structures are highly concentrated. These tissues collectively form the biological basis for the physiological effects produced by stimulating acupoints. Approximately 80% of acupoints are distributed in areas rich in connective tissue, such as the subcutaneous layer, muscle interstices, and regions surrounding bones and joints (110). These areas feature thin epidermal layers, abundant nerve endings, and high mast cell density in the dermis (111, 112). [11C]-carfentanil positron emission tomographic imaging also revealed that the average distance between sensory nerve endings and MCs in acupoint regions is only 15–20 μm, suggesting the potential existence of “neuro-immune synapse” structures (113). This evidence suggests that the unique spatial conformation of acupoints themselves may constitute the morphological basis for acupuncture signal transduction (114, 115), though how these conformations are arranged and whether universal patterns exist remain poorly understood.

Although no reports currently exist on using spatial omics technologies to analyze acupoint structures, this technique has been applied in multiple studies examining the composition of healthy or diseased skin tissues. Ganier et al. (116) found that while skin cell populations are generally conserved, specific cell groups remain unique to different skin locations. For example, mesenchymal cell populations were exclusively present in ear samples, whereas skeletal muscle cell populations were only found in the forehead. Facial skin exhibits higher proportions of T cells, APOD^+^ fibroblasts, POSTN^+^ fibroblasts, and vascular density compared to body skin, while body skin contains more SFRP2^+^ fibroblasts than facial skin. Castillo et al. (117) observed rearrangement of structural elements in psoriatic skin. Compared to healthy or non-lesional skin, lymphatic endothelial cells and vascular endothelial cells in lesional skin are closer to the basement membrane, with fewer immune cells. In mild cases, fibroblasts extend into the superficial dermis near the basement membrane; whereas in moderate-to-severe cases, fibroblasts are displaced into the deep dermis, suggesting that psoriasis severity correlates with the spatial reorganization of specific cells at the dermal-epidermal junction.

In this light, integrating spatial transcriptomics with single-cell sequencing may be pivotal in redefining the acupoint. By generating comparative spatial atlases of established acupoints versus adjacent control regions, researchers can precisely deconstruct the three-dimensional topological configurations including the specific neuro-immune colocalization patterns that define an acupoint’s identity. Furthermore, paired profiling across diverse individuals and disease states aims to validate the “spatiotemporal plasticity” of the acupoint microenvironment. This exploration could ultimately elucidate how the cellular architecture of an acupoint dynamically remodels in response to systemic pathological signals, thereby establishing its molecular anatomical foundation in both health and disease.

### 5.2 Capturing the spatiotemporal dynamics of acupuncture signal initiation

Acupuncture stimulation induces spatiotemporal dynamic remodeling of the local microenvironment, considered the key material basis for generating the sensation of “de qi” (118–120). On one hand, acupuncture stretches the layered network scaffold formed by local connective tissue, inducing conformational changes in connective tissue fibroblasts and cytoskeletal remodeling (121). On the other hand, mechanical force stimulation modulates the biological behavior of mechanosensitive cells such as smooth muscle cells, fibroblasts, endothelial cells, and MCs [e.g., mechanical deformation activates MCs degranulation and promotes the release of signaling molecules like b-FGF and SP from fibroblasts (2, 122)], thereby influencing their functional states and the dynamic equilibrium of the extracellular matrix. However, the direct target cells of acupuncture and the underlying intercellular signaling pathways remain to be thoroughly investigated.

With the advancement of spatial omics technologies, significant breakthroughs have been achieved in addressing similar microenvironmental questions in other fields. Ji et al. (123) employed spatial transcriptomics to analyze the composition of squamous cell carcinoma cells, identifying a tumor-specific population surrounded by a fibro-vascular niche. Ligand-receptor mapping revealed these cells primarily engaged in autocrine and paracrine interactions with cancer-associated fibroblasts, endothelial cells, and macrophages. Liu et al. (124) also utilized spatial mapping to reveal positive correlations between APOE/CD163 tumor-associated macrophages (TAMs) and EMT tumor cells, supporting that TAMs may promote EMT via IGF1-IGF1R interactions in a spatially dependent manner.

To decode the local regulatory mechanisms of acupuncture, future studies should implement a time-series spatial multi-omics design. By mapping the acupoint microenvironment at sequential intervals (e.g., pre-stimulation baseline, immediately post-manipulation during “de qi,” and extended recovery phases), researchers can trace the dynamic trajectory of the mechanotransduction cascade. By employing this longitudinal spatiotemporal approach, researchers can visually capture the transient release of signaling molecules and map early ligand-receptor interactions (e.g., the immediate spatial crosstalk between degranulating MCs and adjacent sensory nerve terminals). This approach may help to elucidate how localized mechanical stimuli are translated into coordinated molecular responses within the targeted tissue architecture.

**FIGURE 5:**
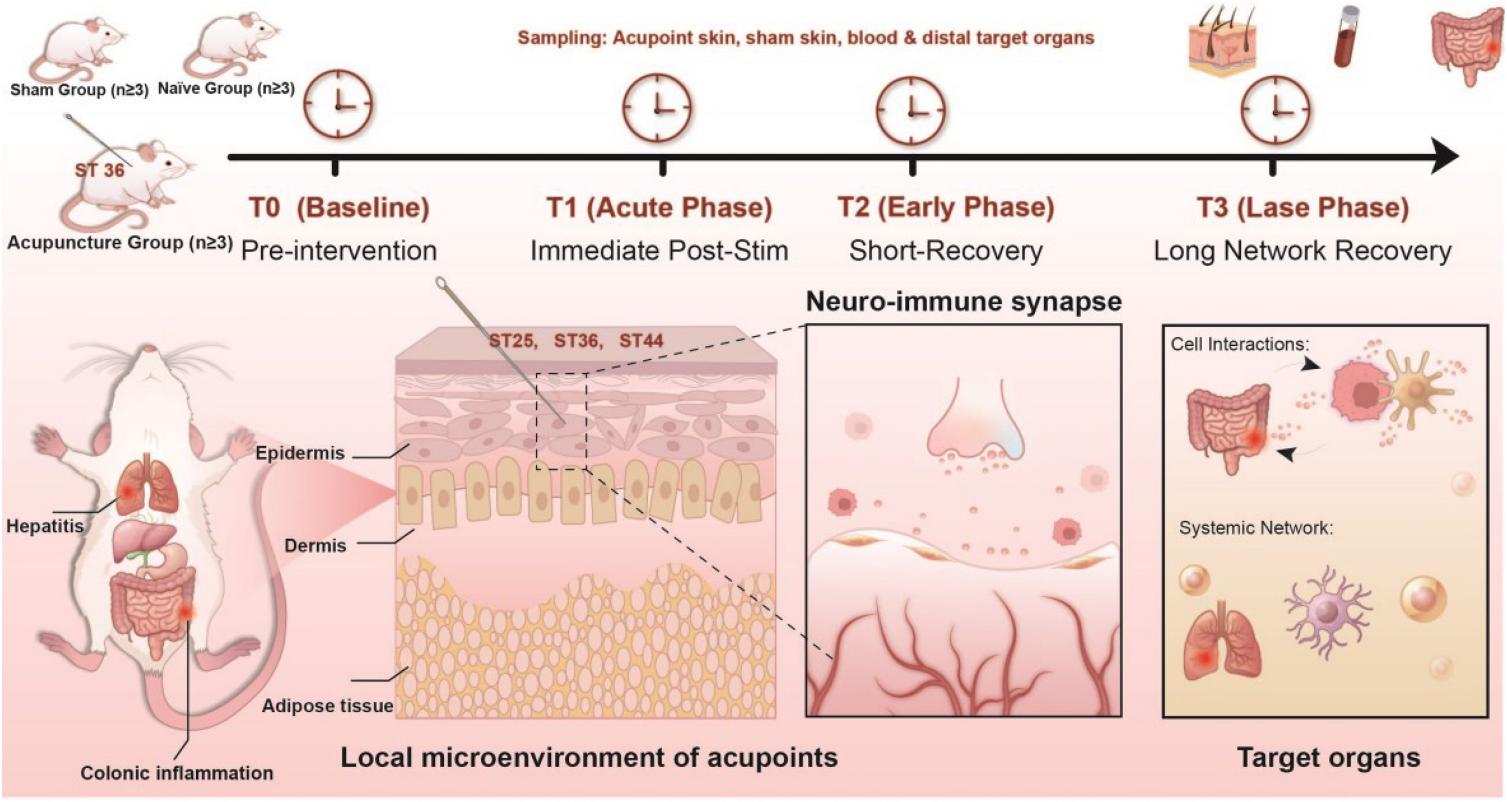
Proposed experimental framework for time-series spatial omics profiling in acupuncture research. This schematic illustrates a multi-scale sampling design across four temporal phases (T0–T3), from the local acupoint microenvironment to systemic target organs. The diagram depicts: (1) a time axis with four sampling phases, with tissue harvesting (skin, blood, and distal target organs) performed at each designated time point using independent animal cohorts to enable destructive sampling; (2) three parallel groups (acupuncture, sham, naïve) at each time point; (3) a magnified view of the skin showing needle insertion at an acupoint (e.g., ST36) and the local neuro-immune synapse; and (4) a distant target organ (e.g., colon) representing the systemic effector site.

The experimental framework outlined in Figure 5 provides a flexible reference for designing such empirical studies. The four temporal phases (T0–T3) are conceptually defined by their biological context rather than fixed numerical windows: T0 establishes baseline (pre-stimulation); T1 captures the acute phase (immediate post-stimulation), reflecting mechanotransduction and local neuro-immune activation; T2 corresponds to an early phase encompassing transcriptional reprogramming and afferent signal propagation; and T3 represents a late phase aimed at detecting systemic network regulation at distal target organs. The specific temporal windows should be empirically optimized for each disease model and stimulation protocol.

Disease models applicable to this design include those in which acupuncture has demonstrated therapeutic efficacy, such as inflammatory bowel disease (e.g., DSS-induced colitis), rheumatoid arthritis, neuropathic pain, and myocardial ischemia, with the distal target organ adjusted accordingly (e.g., colon, joint, spinal cord, or heart). The acupoint–organ pairing should be guided by both traditional meridian theory and modern mechanistic evidence, such as the ST36–vagus–adrenal anti-inflammatory axis identified in the colitis model. For each time point, three parallel groups are recommended: acupuncture, sham (non-acupoint), and naïve (no intervention), using independent cohorts to enable destructive sampling. All tissues should be rapidly harvested, orientation-marked, and snap-frozen to preserve spatial molecular profiles.

### 5.3 Decoding remote network effects through multi-organ spatial mapping

The ability to influence distant pathological sites through interventions at specific acupoints on the body surface is a highly distinctive feature of acupuncture. TCM theory posits that acupoints represent special locations where the “qi” and blood of the internal organs and meridians are projected onto the body surface. Abnormalities at these points reflect pathological conditions within the organs and meridians, and therapeutic effects can be achieved by stimulating them (125). Modern research indicates that acupuncture’s regulatory effects can be mediated through the neuro-endocrine-immune network for distant modulation. Acupuncture activates mechanosensitive sensory neurons on the skin surface, generating molecular signals that travel via reflex arcs and undergo central nervous system processing to exert effects on target sites throughout the body (126). However, the full picture of how these signals are precisely coordinated across the body remains elusive, leaving a significant “black box” in our understanding of acupuncture’s systemic effects.

Common neural transmission pathways include the hypothalamic-pituitary-adrenal axis and the brain-gut axis. Regarding the HPA axis, acupuncture modulates its function by regulating neurotransmitters and their receptors, neuropeptides or hormones and their receptors, as well as microRNA expression, thereby alleviating conditions such as depression, colitis, and surgical trauma (127–129). Regarding the BGA axis, acupuncture controls intestinal inflammation and related neurotransmitter secretion through somatic-autonomic reflex pathways, thereby restoring BGA balance. Concurrently, alterations in the gut microbiota and immune activation signals can be transmitted to the brain via the vagus or sympathetic nerves, establishing bidirectional brain-gut regulation (130). Notably, acupuncture effects often exhibit acupoint specificity. For example, EA stimulation of ST36 on the hindlimb rather than ST25 on the abdomen drives the vagus nerve-adrenal anti-inflammatory axis in mice (131). Further studies revealed that PROKR2-labeled sensory neurons predominantly reside in the deep fascia of the hindlimb, not the abdominal fascia; ablation of these neurons significantly reduced the anti-inflammatory effects of EA (132).

Beyond this, acupuncture can also exert long-distance regulation via interstitial fluid in specific anatomical locations. Recent studies have revealed the presence of vertically distributed interlobular septa at acupoints. These septa connect to surrounding fibrous connective tissues—including the adventitia of arteries and veins, nerves, and skin—forming a vast network that mediates the long-range, directional flow of tissue fluid. This flow pathway closely aligns with traditional meridian pathways (133, 134). For instance, injecting fluorescent tracers into the subcutaneous tissue space at the BL60 acupoint of patients scheduled for amputation allows observation of the tracer within the anatomical layers of the amputated limb post-surgery (135). In human specimens, subcutaneous injection of fluorescent tracers at the LU11 acupoint on the distal thumb tip, powered by an extracardiac automatic compression device, revealed a network of interstitial fluid pathways connecting the thumb tip to cardiac surface tissues upon imaging (133).

**FIGURE 6:**
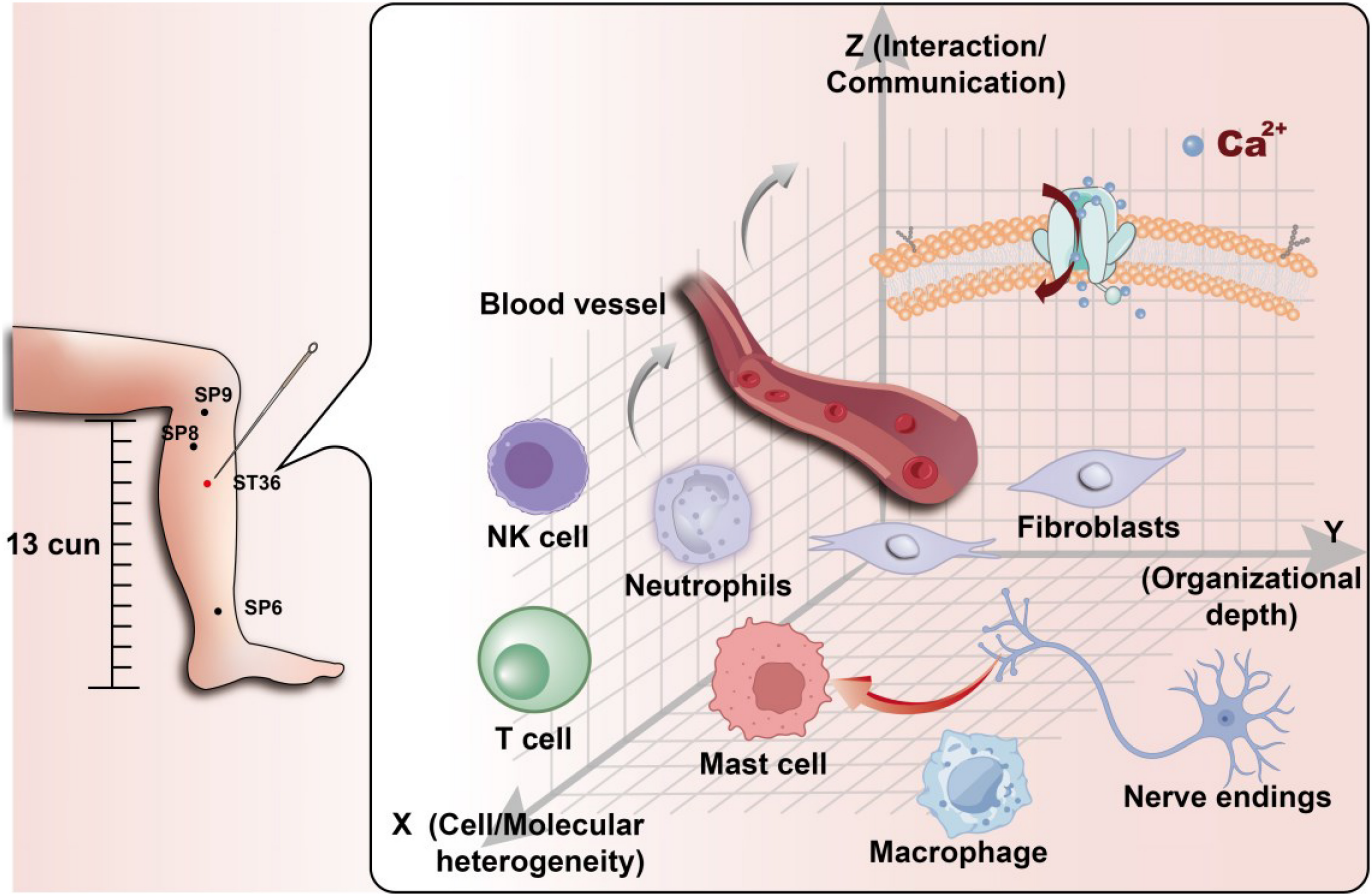
Understanding complex biological systems requires integrating multiple spatial perspectives. This diagram conceptualizes an organ microenvironment as a dynamic interplay of three dimensions: (1) Cell/Molecular heterogeneity (X-axis), capturing diverse cellular components such as mast cells, macrophages, T cells, NK cells, neutrophils, and fibroblasts alongside molecular gradients like Ca^2+^; (2) Organizational depth (Y-axis), detailing the structural organization of these cells around spatial structures like blood vessels and nerve endings; and (3) Interaction/Communication (Z-axis), representing functional ligand-receptor interactions across cell membranes. Spatial omics technologies enable the simultaneous measurement of these dimensions, providing a holistic molecular and cellular map that is crucial for elucidating the mechanisms of systemic therapies. This multidimensional approach demonstrates how acupuncture likely operates by modulating this intricate spatial network from localized needle insertion to broad systemic regulation.

However, the mechanisms underlying acupuncture-mediated cascade regulation across tissues, organs, and even systems remain a “black box.” To definitively bridge this gap, future research requires a synchronous, multi-organ spatial omics mapping strategy (see Figure 6). A robust experimental design would involve simultaneously profiling the spatial transcriptomes of the stimulated acupoint, the intermediate neural relay stations (e.g., dorsal root ganglia, spinal cord, specific brain nuclei), and the distant target organ (e.g., inflamed intestine or ischemic heart) in disease models. This holistic network-level strategy aims to correlate local microenvironmental shifts at the body surface with systemic downstream responses, thereby verifying specific neuro-immune-endocrine axes. By tracking these cross-scale spatial dialogues, research can transition from phenomenological observations to targeted mechanism decoding, offering unprecedented perspectives into acupuncture’s systemic biological principle—where “a single hair moves the entire body.”

### 5.4 Controversies and open questions

To ensure a balanced and objective evaluation of acupuncture biology, existing academic disputes and non-reproducible findings must be explicitly addressed. A major controversy in current acupuncture research revolves around the physiological reality of meridians and the specificity of acupoints (132). While numerous studies report localized anatomical and molecular distinctiveness at acupoints, several large-scale clinical trials and neurophysiological assessments have produced negative or conflicting results, occasionally failing to demonstrate significant therapeutic differences between true acupoints and sham (non-acupoint) needling controls (136). This inconsistency underlines potential variations in needle stimulation intensity, patient baseline states, or the systemic diffusion of placebo effects, which remain unresolved in the field (137).

Furthermore, an overriding limitation of the current literature—and inherently of this predictive review—must be candidly acknowledged: at present, there are no published empirical studies directly applying advanced spatial omics technologies to acupuncture or acupoint biology. Consequently, the spatial principles and molecular architecture proposed herein remain largely conceptual and speculative, representing hypotheses extrapolated from other complex tissue models rather than experimentally validated acupuncture data. Bridging this major gap while rigorously incorporating negative data will be critical to transforming spatial omics from a visionary roadmap into an objective, data-driven framework for acupuncture research.

Finally, establishing a universal or standardized template for specific sampling time points and target tissues remains a critical task for future primary studies. Because optimal temporal windows and anatomical targets will inherently vary depending on the specific animal models and pathological conditions being investigated, future empirical designs must tailor these concrete implementation details based on their explicit biological hypotheses rather than relying on a generalized framework.

## 6 Prospects and challenges

The rapid advancement of spatial omics technologies provides an unprecedented opportunity to transition acupuncture research from phenomenological observations to precise spatial molecular mechanisms (138). Specifically, resolving the neuro-immune microdomains within acupoints (section “5.1 Mapping the spatial architecture of acupoints: from descriptive morphology to molecular cartography”), capturing the transient ligand-receptor interactions during “de qi” (section “5.2 Capturing the spatiotemporal dynamics of acupuncture signal initiation”), and tracing the cross-organ molecular axes underlying remote effects (section “5.3 Decoding remote network effects through multi-organ spatial mapping”) are becoming experimentally tractable. By simultaneously detecting the spatial distribution of proteins, RNA, and metabolites, researchers can decipher the intricate molecular linkages bridging the localized acupoint microenvironment and distant systemic effects across the neuro-endocrine-immune network (77, 139). Furthermore, integrating spatial transcriptomics with single-cell sequencing allows for the precise mapping of cellular heterogeneity, illuminating the synergistic roles of diverse cell types in initiating acupuncture signal transmission (7, 74, 94).

Despite its transformative potential, the application of spatial omics in acupuncture research remains nascent and faces several critical challenges. A primary technological hurdle is the standardization of spatiotemporal data acquisition. Because acupuncture induces highly dynamic and time-sensitive molecular responses, innovative experimental frameworks—such as integrating intravital microscopy with sequential biopsy sampling, or developing organ-on-chip models that replicate acupoint mechanical microenvironments—are required to capture transient signaling events and construct robust multi-omics regulatory networks across multiple time points (138). Without standardized protocols for dynamic tissue sampling, mapping the precise sequence of mechanical-to-chemical signal transduction remains difficult.

Another major challenge lies in the functional plasticity of acupoints. Unlike static anatomical landmarks, acupoint microenvironments dynamically remodel in response to systemic pathology (section “5.1 Mapping the spatial architecture of acupoints: from descriptive morphology to molecular cartography”) and prior stimulation history. This temporal heterogeneity, compounded by inter-individual anatomical variation, necessitates longitudinal sampling designs and the establishment of dynamic, rather than static, population-level atlases. This complexity is well-documented in dermatological research, where spatial transcriptomic studies reveal substantial intra- and inter-individual variation driven by intrinsic skin heterogeneity (75). However, for acupuncture research, the additional dimension of functional state, reflecting the acupoint’s role as a sensor of visceral pathology, introduces unique analytical challenges beyond the anatomical variances typical of skin studies.

To overcome these analytical bottlenecks, future research must pivot toward the integration of artificial intelligence (AI) and advanced computational modeling. By leveraging machine learning algorithms capable of inferring causal regulatory networks from spatial covariance patterns, and graph neural networks that model information flow across the neuro-endocrine-immune axes identified in section “5.3 Decoding remote network effects through multi-organ spatial mapping,” researchers can construct dynamic models that simulate acupuncture’s systemic information transmission pathways (76, 140, 141). Critical algorithmic challenges include disentangling spatial autocorrelation from true biological interaction, integrating multi-scale data from single cells to organ systems, and distinguishing causal mechanotransduction signals from confounding correlations. Addressing these challenges will require domain-specific AI architectures that embed biological priors about neural signaling, immune cell migration, and endocrine feedback loops. Additionally, the continued development of high-resolution three-dimensional spatial profiling and *in vivo* analytical techniques will be pivotal in analyzing thick tissue structures along connective tissue pathways without spatial distortion. Emerging technologies such as mesoscopic photoacoustic imaging and label-free vibrational spectroscopy may enable real-time monitoring of acupoint responses during active stimulation, bridging the gap between static snapshot omics and dynamic physiological processes.

To maintain analytical objectivity and avoid overstated conclusions, the inherent limitations and scope boundaries of the current review must be explicitly articulated. First, while this complementary synthesis focuses heavily on transcriptomic breakthroughs, spatial proteomics and spatial metabolomics remain significantly underrepresented. This technical skew reflects a broader imbalance in current acupuncture methodology; while spatial transcriptomics offers unprecedented genomic depth, it fails to capture functional protein translation or the dynamic flux of local neurochemicals and metabolic signaling molecules (such as lipids, amino acids, and adenosine) within the acupoint microenvironment. Second, profound clinical translation barriers persist as a major bottleneck. The vast majority of discussed spatial omics paradigms rely on invasive, terminal tissue harvesting from small rodent models, which cannot be directly replicated in human clinical trials. Bridging the gap between micro-scale spatial molecular cartography in animals and non-invasive, macro-scale neuroimaging or biomarker monitoring in acupuncture patients remains an unresolved challenge. Acknowledging these technological gaps and cross-species boundaries defines the precise scope of this review, underlining that a truly holistic understanding of acupuncture biology requires the future integration of multi-modal spatial omics platforms coupled with rigorous clinical validation pipelines.

In summary, while significant technical, analytical, and standardization challenges remain, spatial omics offers a revolutionary perspective for decoding the biological essence of acupuncture. Resolving these hurdles through rigorous experimental designs and interdisciplinary collaboration will fully unlock the potential of this cutting-edge technology, ultimately driving the paradigm shift toward precision acupuncture medicine.
